# Supplementary material for: Verifying the concordance between motion corrected and conventional MPRAGE for pediatric morphometric analysis
Source: Front Neurosci. 2025 May 9;19:1534924. doi: 10.3389/fnins.2025.1534924 (PMC12098291; doi:10.3389/fnins.2025.1534924)
Supplement: Supplementary file 1 [file Data_Sheet_1.docx]

**Supplementary Information**

**Verifying the concordance between motion corrected and conventional MPRAGE for pediatric morphometric analysis**

Barat Gal-Er, MSc,^1^ Yannick Brackenier, PhD,^1,2^ Alexandra F. Bonthrone, PhD,^1^ Chiara Casella, PhD,^1,3^ Anthony Price, PhD,^1,4^ Sophie Arulkumaran, PhD,^1^ Andrew T.M. Chew, MD,^1^ Chiara Nosarti, PhD,^1,5^ Michela Cleri, BSc,^2^ Pierluigi Di Cio, BSc,^2^ Alexia Egloff, MD,^1^ Mary A. Rutherford, FRCR,^1^ Jonathan O’Muircheartaigh, PhD,^1,3^ Raphael Tomi-Tricot, PhD,^1,2,6^ Shaihan Malik, PhD^1,2^ Lucilio Cordero-Grande, PhD,^1,2,7^ Joseph V. Hajnal, PhD,^1,2^ Serena J. Counsell, PhD.^1^

1 Centre for the Developing Brain, Research Department of Early Life Imaging, School of Biomedical Engineering and Imaging Sciences, King’s College London, London, UK

2 Research Department of Imaging Physics & Engineering, School of Biomedical Engineering and Imaging Sciences, King's College London, London, UK

3 Department for Forensic and Neurodevelopmental Sciences, Institute of Psychiatry, Psychology and Neuroscience, King’s College London, London, UK

4 Guy’s and St Thomas’ NHS Foundation Trust, London, UK

5 Department of Child and Adolescent Psychiatry, Institute of Psychiatry, Psychology and Neuroscience, King's College London, London, UK

6 MR Research Collaborations, Siemens Healthcare Limited, Camberley, UK

7 Biomedical Image Technologies, ETSI Telecomunicación, Universidad Politécnica de Madrid and CIBER-BNN, ISCIII, Madrid, Spain

**Corresponding author**

Professor Serena J Counsell, Centre for the Developing Brain, School of Biomedical Engineering and Imaging Sciences, King’s College London, London SE1 7EH, UK. Phone: 02071887188. Email: [Serena.Counsell@kcl.ac.uk](mailto:Serena.Counsell@kcl.ac.uk)

**Supplementary Table 1**. Number of datasets requiring manual editing of cortical segmentation.

|  | Datasets requiring manual editing, N (%) |
| --- | --- |
| **DISORDER MPRAGE** |  |
| Motion-free | 2 (10) |
| Motion-corrupt | 4 (20) |
| **Conventional MPRAGE** |  |
| Motion-free | 2 (12) |
| Motion-corrupt | 5 (29) |

**Supplementary Table 2.** ICC and percentage differences between regional brain volumes obtained using conventional MPRAGE and DISORDER data.

|  | Motion-free conventional MPRAGE | | | | Motion-corrupt conventional MPRAGE | | |  |
| --- | --- | --- | --- | --- | --- | --- | --- | --- |
|  | Conventional MPRAGE (x10^3^ mm^3^) | DISORDER  MPRAGE  (x10^3^ mm^3^) | ICC | %D | ConventionalMPRAGE (x10^3^ mm^3^) | DISORDER  MPRAGE  (x10^3^ mm^3^) | ICC | %D |
| Left WM | 210.5  (181.3-246) | 211.7  (186.7-252.6) | 0.96 | 2.1  (-2.5-8.5) | 187.2  (130.3–249) | 212.8  (157.1- 279) | 0.69 | **13.0**  (3.5-20.7) |
| Right WM | 208.5  (180-241.2) | 209.6  (185.9-247.2) | 0.96 | 2.5  (-1.7-7.5) | 187.8  (129 -245.8) | 213.6  (156.6- 272.8) | 0.71 | **13.0**  (2.9-20.2) |
| Left ventral DC | 3.8  (3.1 – 4.4) | 3.7  (3.1 – 4.3) | 0.85 | -6.3  (-10.5-2.5) | 3.7  (3.0 – 4.5) | 3.5  (3.0 – 4.3) | 0.78 | -4.7  (-13-5.6) |
| Right ventral DC | 3.8  (3.2 – 4.7) | 3.6  (3.0 – 4.1) | 0.87 | -5.4  (-10.6-3.3) | 3.6  (3.1-4.5) | 3.5  (2.9 – 4.2) | 0.72 | -5.9  (-15-5.9) |
| Left cerebellar cortex | 56.6  (48.7-73.9) | 53.8  (45.7 – 67.6) | 0.85 | -3.9  (-19.5-2.2) | 59.2  (53.0 – 68.9) | 55.1  (49.5 –69.4) | 0.54 | -3.4  (-15-4.7) |
| Right cerebellar cortex | 56.3  (49.3–72.6) | 54.1  (46.2 – 72.3) | 0.92 | -2.0  (-14.8-4.3) | 58.9  (52.3 – 69.4) | 58.6  (54.1 –71.1) | 0.69 | 0.4  (-8.3-6.8) |
| Left cerebellar WM | 14.0  (11.5–21.8) | 14.6  (11.5 – 26.6) | 0.54 | 2.1  (-12.5-72.2) | 13.8  (10.5 – 17.8) | 16.3  (12.4 –24.3) | 0.19 | 7.7  (-4.4-67.9) |
| Right cerebellar WM | 13.2  (10.7–20.9) | 13.4  (10.7 – 23.1) | 0.47 | -0.5  (-27.7-66.6) | 13.8  (11.2 – 22.0) | 14.2  (11.5 –23.4) | 0.14 | -1.8  (-23.1-51.2) |
| Total left cerebellum | 70.4  (60.1-90.4) | 68.9  (57.7-93.1) | 0.95 | -2.6  (-8.8-3.0) | 73.4  (65.6-84.8) | 72.7  (66.4-86.1) | 0.94 | **0.6**  (-4.0-3.8) |
| Total right cerebellum | 71.1  (60.4-88.6) | 68.8  (57.7-89.0) | 0.95 | -2.1  (-6.2-3.1) | 74.5  (66.5-85.0) | 72.9  (65.7-86.8) | 0.90 | -0.3  (-5.4-5.0) |
| Posterior  CC | 0.84  (0.68–1.07) | 0.87  (0.73 – 1.07) | 0.92 | 1.5  (-4.5-23.3) | 0.8  (0.61 – 1.20) | 0.87  (0.54 – 1.20) | 0.96 | 1.1  (-8.1-16.1) |
| Mid-posterior CC | 0.51  (0.37-0.65) | 0.52  (0.42 -0.66) | 0.84 | 2.6  (-7.3-32.7) | 0.49  (0.39 – 0.66) | 0.50  (0.36 – 0.67) | 0.88 | 2.2  (-17.6-10.7) |
| Central  CC | 0.57  (0.36–0.97) | 0.61  (0.35 – 1.05) | 0.95 | 6.7  (-4.6-30.6) | 0.52  (0.34 – 0.71) | 0.54  (0.31 – 0.68) | 0.66 | -1.0  (-18.3-28.2) |
| Mid-anterior CC | 0.56  (0.35–0.85) | 0.62  (0.35 – 0.91) | 0.90 | 7.4  (-7.7-48.4) | 0.50  (0.34 – 0.86) | 0.49  (0.30 – 0.80) | 0.87 | **-8.7**  (-21.9-5.4) |
| Anterior  CC | 0.82  (0.65 – 1.1) | 0.84  (0.62 – 1.2) | 0.95 | 1.5  (-10-14.7) | 0.83  (0.63 – 1.40) | 0.85  (0.66 – 1.36) | 0.84 | 3.0  (-7.5-19.9) |
| Left LV  Mean (SD) | 4.6  (1.9) | 4.5  (1.9) | 0.99 | -2.9  (-14.5-4.8) | 6.22  (2.5) | 5.90  (2.6) | 0.99 | -3.6  (-21.8-0.7) |
| Right LV  Mean (SD) | 4.4  (1.80) | 4.4  (1.90) | 0.99 | 0.3  (-8.5-5.6) | 5.7  (3.12) | 5.5  (3.20) | 0.99 | -2.7  (-24.2-3.3) |
| Third ventricle  Mean (SD) | 0.79  (0.20) | 0.79  (0.20) | 0.99 | 1.0  (-8.3-9.7) | 0.87  (0.28) | 0.85  (0.25) | 0.97 | -2.7  (-13.4-8.2) |
| Fourth ventricle | 1.66  (1.12–2.90) | 1.74  (1.21 – 2.75) | 0.99 | 1.1  (-10.5-12.1) | 1.81  (1.0 – 3.09) | 1.79  (1.03 – 3.10) | 0.98 | 0.6  (-16.4-10.2) |

MPRAGE volumes are reported as median (range) unless stated otherwise, and percentage differences (%D) are reported as median (95% confidence interval). Percentage differences that are significantly different between motion-free and motion-corrupt MPRAGE data are highlighted in bold. Abbreviations: CC, corpus callosum; DC, diencephalon; LV, lateral ventricle; WM, white matter; SD, standard deviation.

**Results - Sensitivity analyses**

*ICC measures - motion-free conventional MPRAGE and DISORDER data*

After removing CHD participants, ICC measures of agreement between motion-free conventional MPRAGE and DISORDER data for the left total cerebellar volume changed from excellent (ICC 0.95) to good (ICC 0.90). There were no changes for any other brain morphometric measure (Supplementary Table 3).

*ICC measures - motion-corrupt conventional MPRAGE and DISORDER data*

After removing CHD participants, ICC measures of agreement between motion-corrupt conventional MPRAGE and DISORDER data changed from excellent to good for the right CA1 (ICC 0.91 to ICC 0.90) and the left total cerebellar (ICC 0.94 to 0.90) volumes and from moderate to poor for the left CA2 volume (ICC 0.63 to 0.25). Agreement increased from good to excellent for the right total cerebellar volume (ICC 0.90 to 0.92), from moderate to good for left CA4 (ICC 0.57 to 0.78), left total hippocampal (ICC 0.54 to 0.75), right ventral diencephalon (ICC 0.74 to 0.75), left cerebellar cortex (ICC 0.54 to 0.79) and right cerebellar cortex (ICC 0.69 to 0.87) volumes, and from poor to moderate for the right subiculum (ICC 0.31 to 0.53), left CA1 (ICC 0.43 to 0.64), right CA4 (ICC 0.23 to 0.74), right dentate gyrus (ICC 0.29 to 0.62) and left SLRM (ICC 0.45 to 0.67) volumes (Supplementary Table 3).

**Supplementary Table 3.** Comparison of ICC measures and agreement and percentage differences for the whole cohort and for controls only for all morphometric measures assessed in this study. ICC classes of agreement (excellent, good, moderate or poor) that changed after excluding CHD participants are highlighted in bold.

|  | Motion-free conventional MPRAGE | | | | | Motion-corrupt conventional MPRAGE | | | |
| --- | --- | --- | --- | --- | --- | --- | --- | --- | --- |
|  | ICC in the whole cohort, N=20 | ICC in controls only, N=16 | %D in  the whole cohort, N=20 | %D in controls only,  N=16 | ICC in the whole cohort, N=17 | | ICC in controls only,  N=15 | %D in  the whole cohort,  N=17 | %D in controls only,  N=15 |
| Left SA | 0.78  good | 0.76  good | 5.0  (0.7-14) | 5.3  (0.4 -14) | 0.52  moderate | | 0.50  moderate | 22.2  (9.8-33.2) | 22.2  (9.4-33.2) |
| Right SA | 0.81  good | 0.80  good | 5.3  (2.7-12) | 5.6  (3.2 -12) | 0.53  moderate | | 0.54  moderate | 21.2  (8.6-32.4) | 21.2  (8.2-32.5) |
| Left GM | 0.98  excellent | 0.97  excellent | -0.5  (-4.2-5.7) | -0.2  (-4.4-6.1) | 0.73  moderate | | 0.74  moderate | 9.0  (-7.0-29.8) | 9.0  (-7.4-27.3) |
| Right GM | 0.98  excellent | 0.98  excellent | -0.2  (-3.9-4.2) | -0.2  (-4.0-4.3) | 0.74  moderate | | 0.73  moderate | 11.2  (-3.9-26.4) | 11.1  (-4.2-25.6) |
| Left CT | 0.78  good | 0.77  good | -1.4  (-6.9-4.7) | -1.8  (-7.0-5.1) | 0.26  poor | | 0.13  poor | -4.3  (-17.6-3.9) | -4.3  (-17.8-3.4) |
| Right CT | 0.81  good | 0.83  good | -1.7  (-5.5-5.0) | -1.7  (-5.0-3.6) | 0.09  poor | | 0.06  poor | -5.2  (-15.4-3.7) | -5.2  (-15.6-3.3) |
| Left mean curvature | 0.77  good | 0.78  good | 2.4  (-4.6-6.7) | 2.0  (-5.2-6.0) | 0.12  poor | | 0.14  poor | -9.1  (-16.2-0.5) | -9.1  (-16.4-0.8) |
| Right mean curvature | 0.76  good | 0.81  good | 1.9  (-3.7-7.1) | 1.2  (-4.0-5.6) | 0.16  poor | | 0.15  poor | -10.1  (-14.7-2.3) | -10.1  (-14.7-2.3) |
| Left LGI | 0.92  excellent | 0.94  excellent | -0.1  (-3.6-3.5) | -0.2  (-2.0-3.7) | 0.45  poor | | 0.46  poor | 7.6  (0.2-12.7) | 7.1  (-0.1-12.7) |
| Right LGI | 0.92  excellent | 0.94  excellent | 0.2  (-3.7-4.0) | 0.4  (-1.2-4.1) | 0.48  poor | | 0.45  poor | 9.1  (0.1-17.2) | 7.9  (0.1- 17.0) |
| Left  Thalamus | 0.96  excellent | 0.96  excellent | -1.8  (-6.2-4.1) | -2.3  (-6.3-3.7) | 0.98  excellent | | 0.98  excellent | -1.4  (-6.2-2.7) | -1.6  (-6.3-2.0) |
| Right Thalamus | 0.96  excellent | 0.95  excellent | -2.3  (-6.6-1.7) | -3.0  (-6.7-0.7) | 0.97  excellent | | 0.97  excellent | -2.4  (-6.3-2.1) | -2.5  (-6.4-1.3) |
| Left Caudate | 0.93  excellent | 0.92  excellent | -2.4  (-11.4-7.) | -2.4  (-12.2-7.3) | 0.75  good | | 0.89  good | 3.2  (-12.5-16.1) | 4.1  (-7.6-16.6) |
| Right Caudate | 0.93  excellent | 0.92  excellent | -3.2  (-16-6.5) | -3.2  (-16.5-7.9) | 0.62  moderate | | 0.74  moderate | 4.6  (-14.7-13.8) | 5.6  (-10.3-14) |
| Left Putamen | 0.75  good | 0.81  good | -8.1  (-16- -2.3) | -8.1  (-16.4- -2) | 0.88  good | | 0.82  good | -2.3  (-11.0-20.1) | -2.3  (-11.2-9.7) |
| Right Putamen | 0.76  good | 0.79  good | -7.8  (-15- -3.5) | -8.4  (-16- -5.1) | 0.85  good | | 0.87  good | -5.9  (-11.8-10.5) | -6.3  (-11.9-9.7) |
| Left GP | 0.82  good | 0.86  good | -2.3  (-7.7-4.6) | -2.3  (-7.9-4.9) | 0.77  good | | 0.89  good | -2.5  (-10.6-8.2) | -2.7  (-10.7-2.9) |
| Right GP | 0.89  good | 0.89  good | -6.3  (-10.8-2.3) | -7.0  (-10.9-2.9) | 0.88  good | | 0.83  good | -0.9  (-7.5-8.1) | -1.0  (-7.6-3.7) |
| Left Amygdala | 0.38  poor | 0.40  poor | 10.0  (-6.1-43.3) | 9.6  (-7.1-42.7) | 0.26  poor | | 0.36  poor | 22.2  (-2.4-97.9) | 13.9  (-2.9-99) |
| Right Amygdala | 0.65  moderate | 0.62  moderate | 7.6  (-8.5-48.9) | 7.8  (-9.6-50.3) | 0.42  poor | | 0.45  poor | 24.2  (-10.2-47.3) | 25.2  (-10.8-47.8) |
| Left NA | 0.64  moderate | 0.56  moderate | 13.0  (-0.5-38.2) | 15.6  (0.1-40.7) | 0.10  poor | | 0.17  poor | 36.3  (-4.1-105.9) | 38.2  (3.6-107) |
| Right NA | 0.47  poor | 0.36  poor | 14.0  (-12-78.2) | 17.7  (-13-78.7) | 0.36  poor | | 0.44  poor | 36.3  (-29.8-86.4) | 36.3  (-30.8-88) |
| Brainstem | 0.93  excellent | 0.92  excellent | -2.6  (-7.8-8.7) | -2.9  (-7.9 -9.1) | 0.85  good | | 0.89  good | -4.2  (-9.6-5.9) | -4.2  (-8.7-6.3) |
| Left subiculum | 0.74  moderate | 0.67  moderate | -6.6  (-18.1-7.2) | -8.2  (-18.3-7.9) | 0.17  poor | | 0.45  poor | -3.6  (-20.6-95.4) | -3.6  (-20.6-95.5) |
| Right subiculum | 0.81  good | 0.79  good | -6.0  (-19.0-1.6) | -6.5  (-20- -1.2) | 0.31  poor | | **0.53**  **moderate** | -2.0  (-31-29.2) | -2.0  (-32.3-29.3) |
| Left CA1 | 0.99  excellent | 0.98  excellent | 1.8  (-3.6-6.0) | 0.4  (-3.8-6.3) | 0.43  poor | | **0.64**  **moderate** | 4.0  (-11.7-86.7) | 3.0  (-11.9-89.3) |
| Right CA1 | 0.96  excellent | 0.97  excellent | 0.7  (-3.2-7.1) | 0.5  (-2.1-7.1) | 0.91  excellent | | **0.90**  **good** | 2.9  (-8.3-13.6) | 2.9  (-8.4-10.3) |
| Left CA2 | 0.65  moderate | 0.68  moderate | -3.5  (-19.8-28.4) | -0.8  (-19.7-29) | 0.63  moderate | | **0.25**  **poor** | -17.3  (-68.7-41.2) | -19.9  (-68.7-39.5) |
| Right CA2 | 0.82  good | 0.75  good | -6.4  (-26.3-19.9) | -5.7  (-26.7-9.4) | 0.90  good | | 0.80  good | 14.0  (-9.7-75.2) | 14.0  (-10.0-77.3) |
| Left CA3 | 0.86  good | 0.85  good | -6.9  (-15.9-10.1) | -6.9  (-12.4-11) | 0.11  poor | | 0.20  poor | -5.8  (-60.3-30.1) | -7.9  (-61.3-30.3) |
| Right CA3 | 0.90  good | 0.90  good | -4.3  (-14.0-17.6) | -3.5  (-13.7-18) | 0.62  moderate | | 0.70  moderate | 10.6  (-8.3-35.3) | 6.8  (-8.4-35.6) |
| Left CA4 | 0.94  excellent | 0.91  excellent | -5.2  (-25.6-24.8) | -5.2  (-27-26.1) | 0.57  moderate | | **0.78**  **good** | -3.1  (-34-137.3) | -3.1  (-35.4-139) |
| Right CA4 | 0.95  excellent | 0.94  excellent | -7.0  (-18.5-2.6) | -6.3  (-17.6-3.0) | 0.23  poor | | **0.74**  **moderate** | -4.7  (-36.2-38.8) | -3.0  (-31.1-39.2) |
| Left DG | 0.95  excellent | 0.95  excellent | -2.9  (-12.0-1.4) | -2.9  (-12.5-1.3) | 0.38  poor | | 0.48  poor | -0.5  (-8.2-112.4) | -0.5  (-8.3-113) |
| Right DG | 0.95  excellent | 0.97  excellent | -1.3  (-11.2-6.3) | 0.4  (-6.9-6.6) | 0.29  poor | | **0.62**  **moderate** | 10.1  (-6.0-66.8) | 10.1  (-6.0-67.1) |
| Left SRLM | 0.96  excellent | 0.95  excellent | -1.8  (-9.2-2.3) | -1.8  (-9.4-2.3) | 0.45  poor | | **0.67**  **moderate** | -0.6  (-11.1-103.) | -0.6  (-11.6-105) |
| Right SRLM | 0.96  excellent | 0.96  excellent | -2.7  (-7.3-3.5) | -2.7  (-7.4-1.4) | 0.84  good | | 0.89  good | -2.2  (-8.4-23.4) | -2.2  (-8.4-23.9) |
| Left total hippocampus | 0.96  excellent | 0.95  excellent | -2.1  (-8.0-0.5) | -1.9  (-8.3-0.5) | 0.54  moderate | | **0.75**  **good** | -2.6  (-11.7-21.4) | -4.0  (-12.1-21.9) |
| Right total hippocampus | 0.96  excellent | 0.96  excellent | -2.3  (-6.1-0.6) | -2.3  (-6.2-0.7) | 0.88  good | | 0.89  good | 0.3  (-5.9-17.9) | 0.3  (-6.0-18.0) |
| Left WM | 0.96  excellent | 0.95  excellent | 2.1  (-2.5-8.5) | 2.1  (-2.8 -8.9) | 0.69  moderate | | 0.69  moderate | 13.0  (3.5-20.7) | 12.7  (3.2-20.7) |
| Right WM | 0.96  excellent | 0.95  excellent | 2.5  (-1.7-7.5) | 2.6  (-1.1-7.8) | 0.71  moderate | | 0.74  moderate | 13.0  (2.9-20.2) | 12.7  (2.7-20.3) |
| Left ventral DC | 0.85  good | 0.86  good | -6.3  (-10.5-2.5) | -6.8  (-10.6-3.0) | 0.78  good | | 0.87  good | -4.7  (-13-5.6) | -5.0  (-12.9-5.7) |
| Right ventral DC | 0.87  good | 0.88  good | -5.4  (-10.6-3.3) | -6.1  (-10.7-3.0) | 0.72  moderate | | **0.75**  **good** | -5.9  (-15-5.9) | -6.3  (-15.3-2.4) |
| Left cerebellar cortex | 0.85  good | 0.87  good | -3.9  (-19.5-2.2) | -4.3  (-17.2-2.3) | 0.54  moderate | | **0.79**  **good** | -3.4  (-15-4.7) | -3.5  (-15.4-3.0) |
| Right cerebellar cortex | 0.92  excellent | 0.92  excellent | -2.0  (-14.8-4.3) | -2.6  (-15.1-4.8) | 0.69  moderate | | **0.87**  **good** | 0.4  (-8.3-6.8) | 0.4  (-5.8-7.0) |
| Left cerebellar WM | 0.54  moderate | 0.51  moderate | 2.1  (-12.5-72.2) | -0.1  (-13.7-75) | 0.19  poor | | 0.34  poor | 7.7  (-4.4-67.9) | 9.6  (-4.6-68.1) |
| Right cerebellar WM | 0.47  poor | 0.35  poor | -0.5  (-27.7-66.6) | -0.5  (-29.5-67) | 0.14  poor | | 0.25  poor | -1.8  (-23.1-51.2) | -2.8  (-24.3-28.3) |
| Left total cerebellum | 0.95  excellent | **0.90**  **good** | -2.6  (-8.8-3.0) | -3.0  (-5.7-3.3) | 0.94  excellent | | **0.90**  **good** | 0.6  (-4.0-3.8) | 0.6  (-4.1-3.0) |
| Right total cerebellum | 0.95  excellent | 0.91  excellent | -2.1  (-6.2-3.1) | -2.5  (-5.7-3.0) | 0.90  good | | **0.92**  **excellent** | -0.3  (-5.4-5.0) | -0.4  (-5.4-3.2) |
| Posterior CC | 0.92  excellent | 0.93  excellent | 1.5  (-4.5-23.3) | 2.2  (-4.6-18.1) | 0.96  excellent | | 0.95  excellent | 1.1  (-8.1-16.1) | 2.2  (-8.5-16.4) |
| Mid-posterior CC | 0.84  good | 0.81  good | 2.6  (-7.3-32.7) | 2.6  (-8.0-34.8) | 0.88  good | | 0.89  good | 2.2  (-17.6-10.7) | 2.2  (-17.6-9.7) |
| Central CC | 0.95  excellent | 0.92  excellent | 6.7  (-4.6-30.6) | 6.7  (-0.3-30.7) | 0.66  moderate | | 0.74  moderate | -1.0  (-18.3-28.2) | -1.0  (-18.3-28.4) |
| Mid-anterior CC | 0.90  good | 0.90  good | 7.4  (-7.7-48.4) | 7.4  (-7.0-38.6) | 0.87  good | | 0.88  good | -8.7  (-21.9-5.4) | -8.7  (-21.9-5.4) |
| Anterior CC | 0.95  excellent | 0.92  excellent | 1.5  (-10-14.7) | 2.2  (-10.1-16) | 0.84  good | | 0.87  good | 3.0  (-7.5-19.9) | 2.5  (-7.5-20.4) |
| Left LV | 0.99  excellent | 0.99  excellent | -2.9  (-14.5-4.8) | -3.4  (-15.7-4.2) | 0.99  excellent | | 0.99  excellent | -3.6  (-21.8-0.7) | -3.6  (-21.3-0.9) |
| Right LV | 0.99  excellent | 0.99  excellent | 0.3  (-8.5-5.6) | -0.3  (-8.7-5.7) | 0.99  excellent | | 0.99  excellent | -2.7  (-24.2-3.3) | -2.7  (-24.2-3.4) |
| Third ventricle | 0.99  excellent | 0.98  excellent | 1.0  (-8.3-9.7) | -0.3  (-9.0-5.7) | 0.97  excellent | | 0.98  excellent | -2.7  (-13.4-8.2) | -2.7  (-13.6-8.1) |
| Fourth ventricle | 0.99  excellent | 0.99  excellent | 1.1  (-10.5-12.1) | -0.1  (-10.9-9.3) | 0.98  excellent | | 0.99  excellent | 0.6  (-16.4-10.2) | 0.6  (-16.7-7.4) |

Percentage differences (%D) are reported as median (estimated 95% confidence intervals). Abbreviations: CC, corpus callosum; CA, cornu ammonius; CT, cortical thickness; DC, diencephalon; DG, dentate gyrus; GM, grey matter; GP, globus pallidus; LGI, local gyrification index; LV, lateral ventricles; NA, nucleus accumbens; SA, surface area; SRLM, stratum radiatum lacunosum and moleculare; WM, white matter.

**Supplementary Table 4.** Percentage difference [(Left-Right)/(Left + Right)]∗ 100 between left and right subcortical grey matter volumes, shown separately for DISORDER and (i) motion-free and (ii) motion-corrupt conventional MPRAGE data.

|  | Motion-free conventional  MPRAGE | | Motion-corrupt conventional MPRAGE | |
| --- | --- | --- | --- | --- |
|  | Conventional MPRAGE | DISORDER MPRAGE | Conventional MPRAGE | DISORDER MPRAGE |
| Thalamus | 1.5  (-0.4 – 3.6) | 1.8  (-0.2 – 3.8) | 1.5  (-1.4 – 3.7) | 2.1  (0.6 – 3.9) |
| Caudate nucleus | -2.6  (-8.0 – 6.0) | -1.0  (-4.9 – 7.8) | **-6.5**  (-13.8 - -1.8) | -2.5  (-4.6 – 9.0) |
| Putamen | 0.5  (-3.6 – 4.3) | 0.7  (-2.1 – 5.3) | 0.2  (-5.8 – 5.3) | 2.0  (-7.0 – 6.8) |
| Globus pallidus | 1.1  (-2.2 – 4.0) | 0.6  (-2.4 – 3.8) | 2.2  (-1.9 – 5.6) | 0.89  (-2.6 – 4.7) |
| Amygdala | 3.6  (-8.7 – 17.2) | 4.4  (-3.9 – 11.6) | 6.6  (-14.0 – 20.3) | 6.4  (-5.1 – 16.6) |
| Nucleus accumbens | 10.3  (-9.2 – 29.7) | 10.2  (-1.3 – 21.9) | 7.5  (-21.2 – 30.9) | 12.3  (-3.6 – 28.9) |

Percentage differences are reported as median (95% confidence interval). Percentage differences that are significantly different between conventional and DISORDER MPRAGE data are highlighted in bold.

**Supplementary Table 5.** Percentage difference [(Left-Right)/(Left + Right)] ∗ 100 between left and right hippocampal volumes, shown separately for DISORDER and (i) motion-free and (ii) motion-corrupt conventional MPRAGE data.

|  | Motion-free conventional  MPRAGE | | Motion-corrupt conventional MPRAGE | |
| --- | --- | --- | --- | --- |
|  | Conventional MPRAGE | DISORDER MPRAGE | Conventional MPRAGE | DISORDER MPRAGE |
| Subiculum | 1.0  (-4.7 – 10.7) | 0.16  (-5.8 – 10.7) | -5.6  (-31.1 – 9.8) | -0.5  (-10.4 – 7.7) |
| CA1 | -2.8  (-11.6 – 1.9) | -3.1  (-10.5 – 0.3) | -4.7  (-31.3 – 5.6) | -2.1  (-8.8 – 2.5) |
| CA2 | 6.7  (-3.4 – 29.4) | 8.6  (-1.8 – 22.9) | **18.8**  (-27.4 – 61.1) | 6.6  (-18.3 – 22.7) |
| CA3 | 11.1  (1.3 – 23.7) | 8.9  (-0.6 – 17.0) | **13.4**  (-10.9 – 41.8) | 7.8  (-9.8 – 18.1) |
| CA4 | -12.5  (-28.5 – 7.9) | -12.1  (-22.5 – 3.2) | -13.4  (-45.1 – 13.6) | -11.6  (-32.1 – 15.6) |
| Dentate gyrus | -0.1  (-5.0 – 9.2) | -0.5  (-8.7 – 6.0) | 2.8  (-27.1 – 12.9) | -1.0  (-6.6 – 6.8) |
| SRLM | -1.7  (5.4 – 1.8) | -2.0  (-4.5 – 1.1) | -3.4  (-29.3 – 5.0) | -1.4  (-8.6 – 2.9) |
| Hippocampus | -0.8  (-3.3 – 3.4) | -1.0  (-2.7 – 1.9) | -0.6  (-18.0 – 6.1) | -0.9  (-7.0 – 2.1) |

Percentage differences are reported as median (95% confidence interval). Abbreviations: CA, cornu ammonius; SRLM, stratum radiatum lacunosum and moleculare. Percentage differences that are significantly different between conventional and DISORDER MPRAGE data are highlighted in bold.

**Supplementary Table 6.** Percentage difference [(Left-Right)/(Left + Right)] ∗ 100 between left and right cortical measures, shown separately for DISORDER and (i) motion-free and (ii) motion-corrupt conventional MPRAGE data.

|  | Motion-free conventional MPRAGE | | Motion-corrupt conventional MPRAGE | |
| --- | --- | --- | --- | --- |
|  | Conventional MPRAGE | DISORDER MPRAGE | Conventional MPRAGE | DISORDER MPRAGE |
| Surface area | 0.2  (-1.0 – 1.1) | 0.1  (-1.5 – 1.7) | 0.1  (-3.8 – 3.1) | 0.1  (-1.0 – 2.1) |
| Grey matter volume | 0.4  (-0.5 – 1.4) | 0.5  (-0.7 – 1.2) | **1.0**  (-1.5 – 8.8) | 0.05  (-1.1 – 1.9) |
| Cortical thickness | 0.3  (-0.6 – 1.5) | 0.6  (-1.1 – 1.1) | 0.1  (-1.6 – 1.5) | 0.01  (-1.6 – 1.5) |
| Mean curvature | 0.2  (-1.9 – 1.8) | 0.1  (-1.9 – 0.8) | 0.2  (-1.7 – 1.6) | 0.2  (-1.5 – 2.5) |
| Gyrification index | 0.1  (-1.2 – 1.3) | 0.2  (-1.6 – 1.3) | 0.6  (-0.8 – 2.7) | 0.5  (-0.8 – 2.0) |

Percentage differences are reported as median (95% confidence interval). Percentage differences that are significantly different between conventional and DISORDER MPRAGE data are highlighted in bold.

**Supplementary Table 7.** Percentage difference [(Left-Right)/(Left + Right)] ∗ 100 between left and right regional brain volumes, shown separately for DISORDER and (i) motion-free and (ii) motion-corrupt conventional MPRAGE data.

|  | Motion-free conventional MPRAGE | | Motion-corrupt conventional MPRAGE | |
| --- | --- | --- | --- | --- |
|  | Conventional MPRAGE | DISORDER MPRAGE | Conventional MPRAGE | DISORDER MPRAGE |
| White matter | 0.4  (-0.8 – 0.9) | 0.3  (-1.2 – 2.7) | 0.2  (-1.2 – 2.7) | 0.3  (-0.4 – 2.7) |
| Cerebellar white matter | 1.5  (-14.5 – 7.2) | 4.2  (-9.2 – 9.0) | **-1.1**  (-16.7 – 3.9) | 4.4  (-15.3 – 6.5) |
| Cerebellar cortex | -0.03  (-2.2 – 3.6) | -0.5  (-6.6 – 2.9) | -0.3  (-1.8 – 3.3) | -1.0  (-4.5 – 2.6) |
| Total cerebellum | 0.4  (-2.3 – 2.6) | 0.1  (-3.2 – 3.2) | -0.6  (-2.1 – 1.2) | 0.03  (2.8 – 2.6) |
| Ventral DC | 0.3  (-2.5 – 3.0) | 0.5  (-2.6 – 3.0) | 0.1  (-3.2 – 2.9) | 0.9  (-1.2 – 1.3) |
| Lateral ventricles | 6.2  (-17.4 – 23.0) | 4.4  (-18.9 – 23.1) | 1.9  (-7.5 – 22.4) | 6.9  (-8.7 – 19.6) |

Percentage differences are reported as median (95% confidence interval). Abbreviations: DC, diencephalon. Percentage differences that are significantly different between conventional and DISORDER MPRAGE data are highlighted in bold.

**Supplementary Figure Legends**

**Supplementary Figure 1.** MPRAGE data before and after Gibbs unringing with mrdeGibbs3D. The top row shows a motion-corrupt conventional MPRAGE acquisition (Ai, Bi) and the bottom row shows the DISORDER MPRAGE acquisition (Aii, Bii) from the same individual [Female, 8 years]. Arrows indicate regions where Gibbs ringing artifacts are diminished after mrdeGibbs3D.

**Supplementary Figure 2.** Percentage difference between regional brain volumes obtained using DISORDER vs motion-free conventional MPRAGE data and DISORDER vs motion-corrupt conventional MPRAGE data. Abbreviations: CC, corpus callosum. ns indicates a non-significant difference, **** indicates a significant difference at the pFDR < 0.0001 level, *** indicates a significant difference at the pFDR < 0.001 level.

**Supplementary Figure 3.** Percentage difference between left and right subcortical grey matter volumes, shown separately for DISORDER and (i) motion-free and (ii) motion-corrupt conventional MPRAGE data. ns indicates a non-significant difference and *** indicates a significant difference at the pFDR < 0.001 level.

**Supplementary Figure 4.** Percentage difference between left and right hippocampal volumes, shown separately for DISORDER and (i) motion-free and (ii) motion-corrupt conventional MPRAGE data. ns indicates a non-significant difference and * indicates a significant difference at the pFDR < 0.05 level.

**Supplementary Figure 5.** Percentage difference between left and right cortical measures, shown separately for DISORDER and (i) motion-free and (ii) motion-corrupt conventional MPRAGE data. ns indicates a non-significant difference and * indicates a significant difference at the pFDR < 0.05 level.

**Supplementary Figure 6.** Percentage difference between left and right regional brain volumes, shown separately for DISORDER and (i) motion-free and (ii) motion-corrupt conventional MPRAGE data. ns indicates a non-significant difference and ** indicates a significant difference at the pFDR < 0.01 level.

**Supplementary Figures**

**
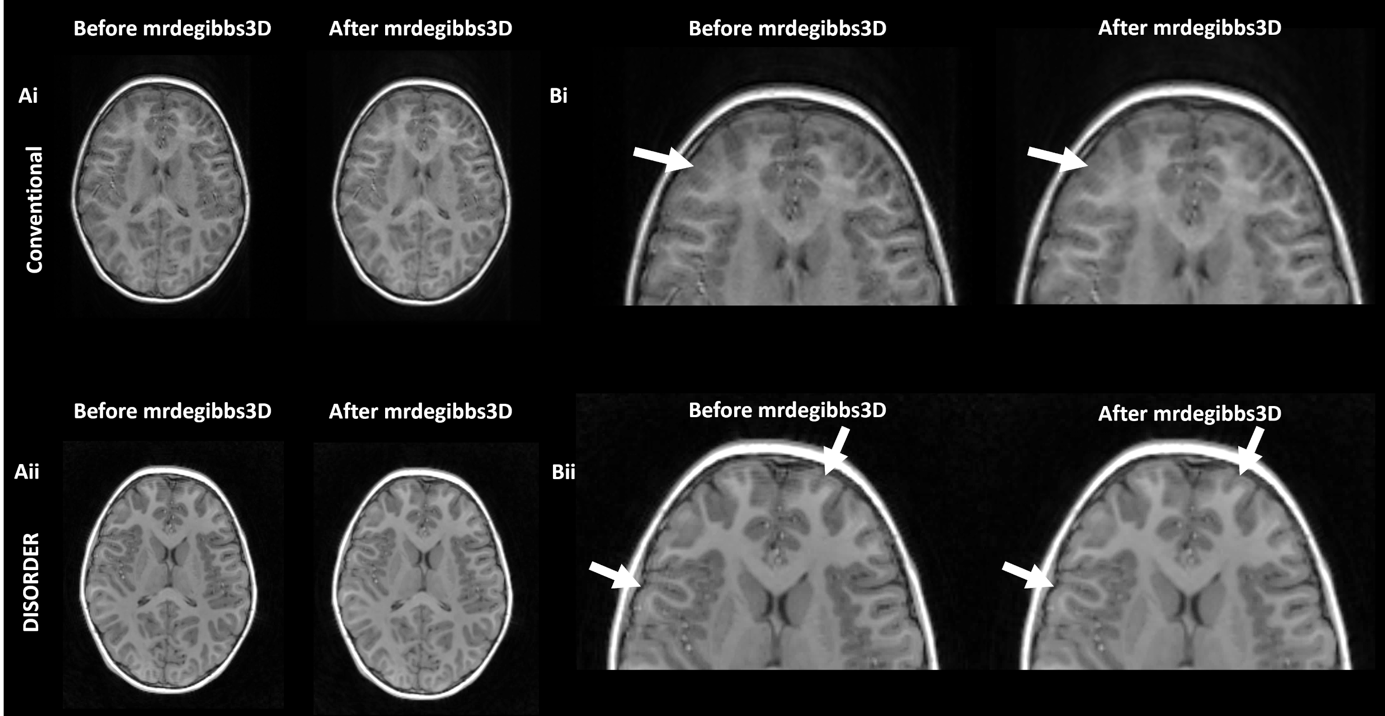
**

**Supplementary Figure 1.** MPRAGE data before and after Gibbs unringing with deGibbs3D. The top row shows a motion-corrupt conventional MPRAGE acquisition (Ai, Bi) and the bottom row shows the DISORDER MPRAGE acquisition (Aii, Bii) from the same individual [Female, 8 years]. Arrows indicate regions where Gibbs ringing artifacts are diminished after deGibbs3D.

**
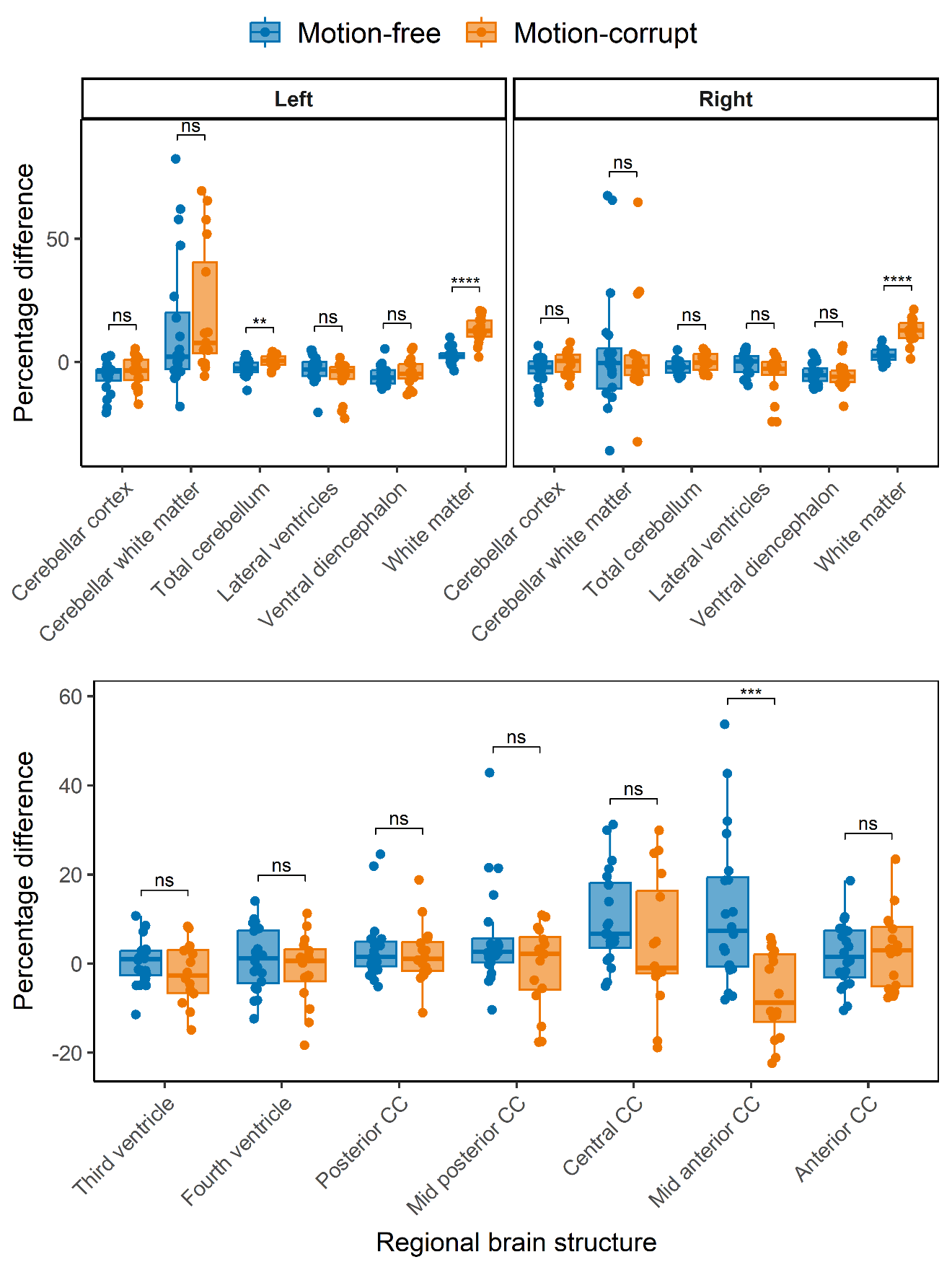
**

**Supplementary Figure 2.** Percentage difference between regional brain volumes obtained using DISORDER vs motion-free conventional MPRAGE data and DISORDER vs motion-corrupt conventional MPRAGE data. Abbreviations: CC, corpus callosum. ns indicates a non-significant difference, **** indicates a significant difference at the pFDR < 0.0001 level, *** indicates a significant difference at the pFDR < 0.001 level.

**
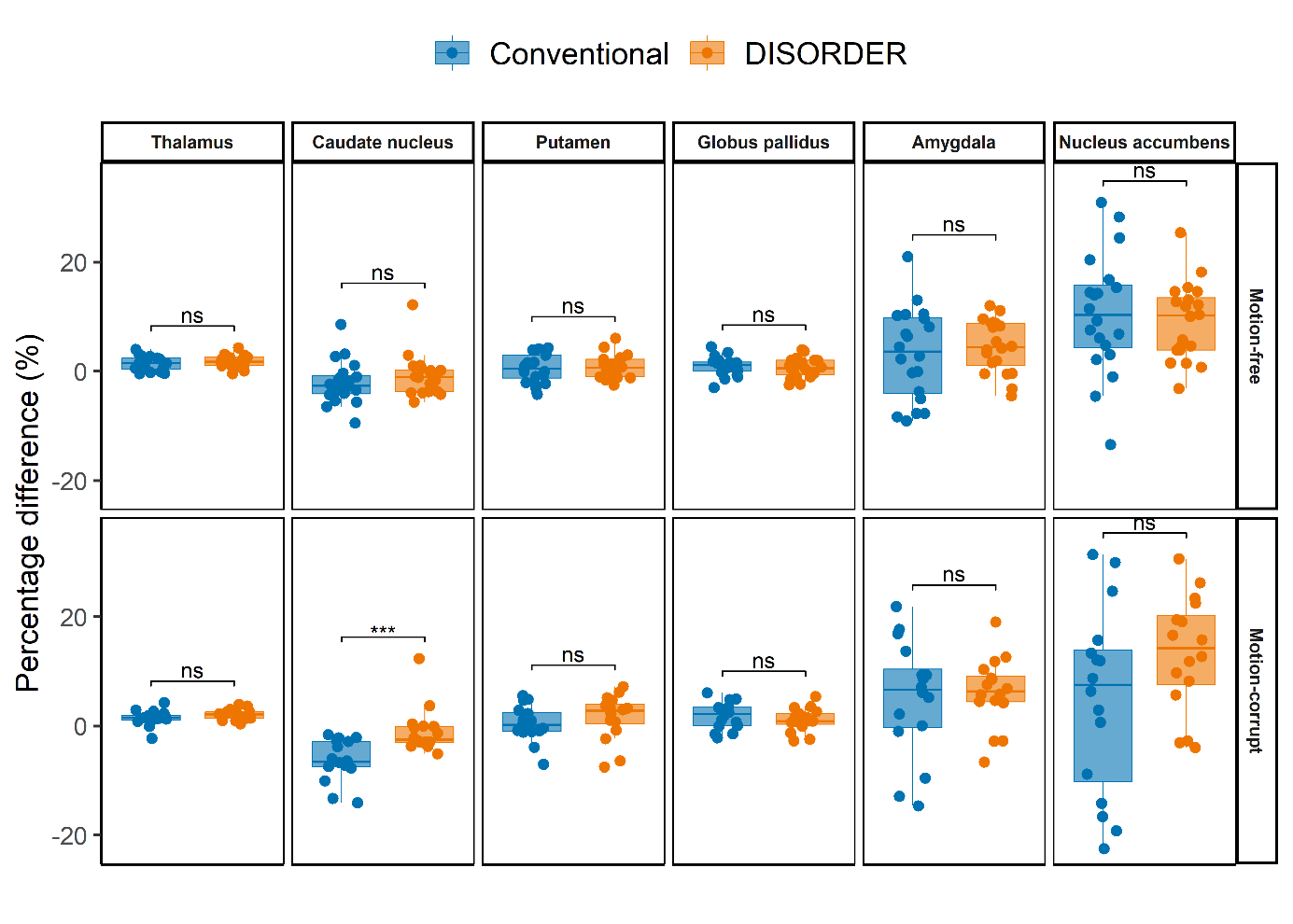
**

**Supplementary Figure 3.** Percentage difference between left and right subcortical volumes, shown separately for DISORDER and (i) motion-free and (ii) motion-corrupt conventional MPRAGE data. ns indicates a non-significant difference and *** indicates a significant difference at the pFDR < 0.001 level.


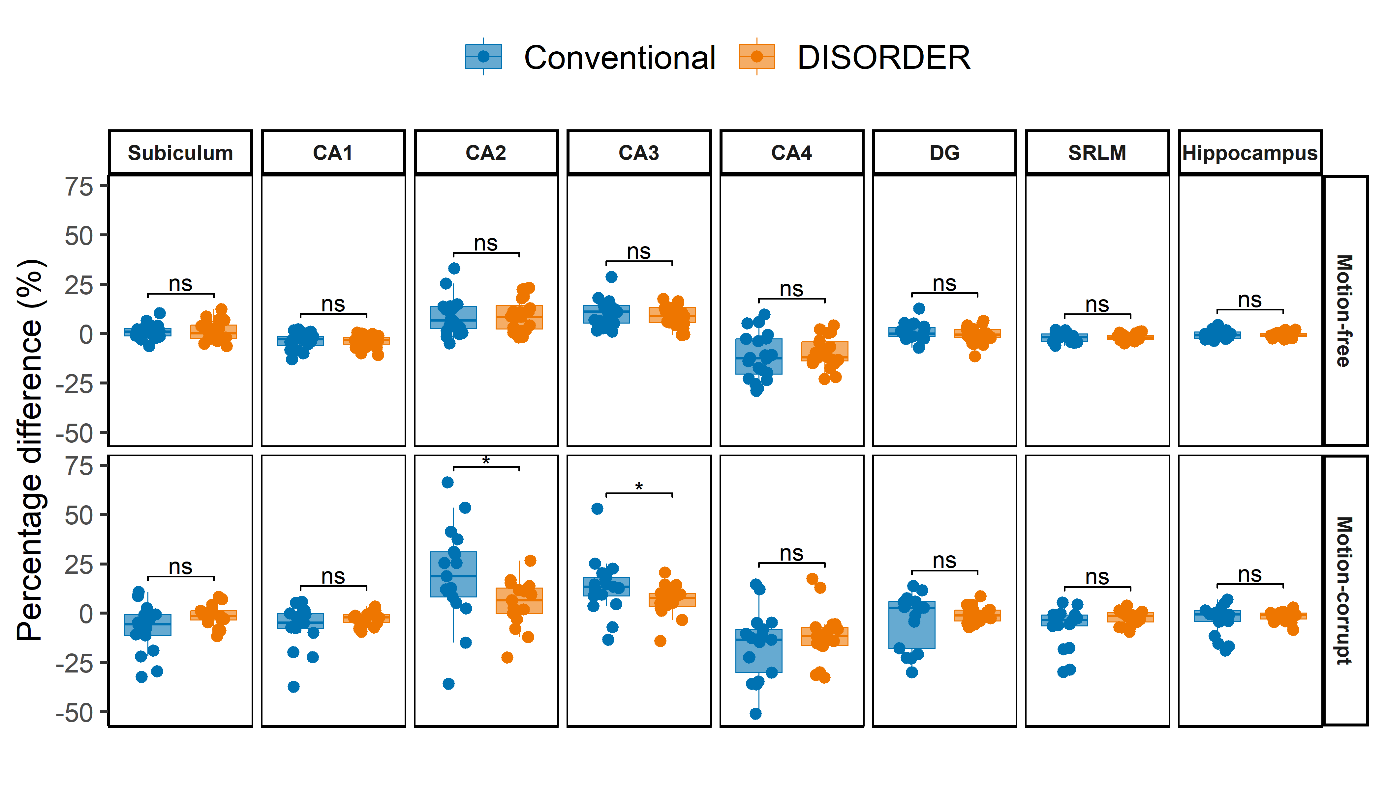


**Supplementary Figure 4.** Percentage difference between left and right hippocampal volumes, shown separately for DISORDER and (i) motion-free and (ii) motion-corrupt conventional MPRAGE data. Abbreviations: CA, cornus ammonis; DG, dentate gyrus; SRLM, stratum radiatum lacunosum and moleculare. ns indicates a non-significant difference and * indicates a significant difference at the pFDR < 0.05 level.


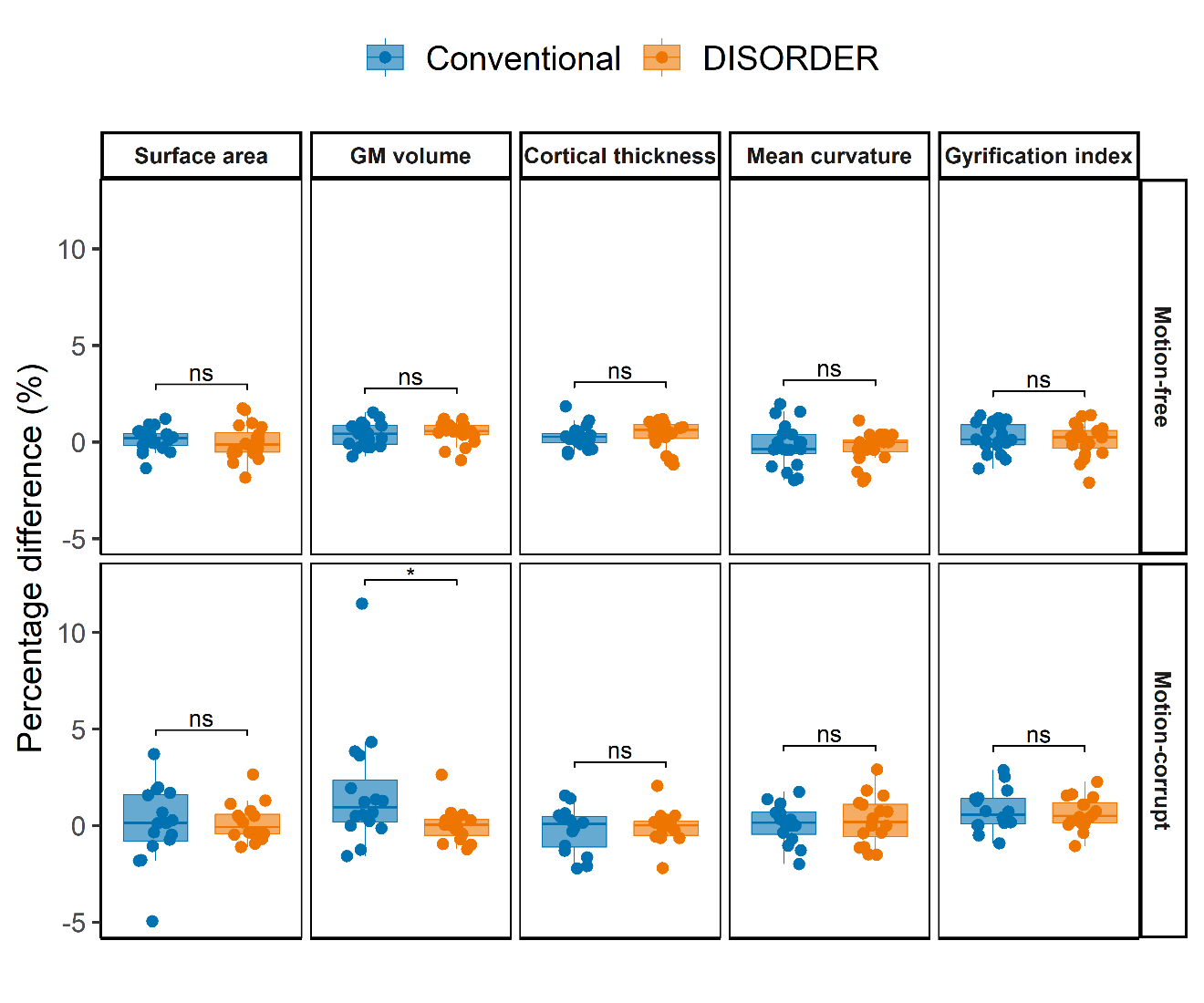


**Supplementary Figure 5.** Percentage difference between left and right cortical measures, shown separately for DISORDER and (i) motion-free and (ii) motion-corrupt conventional MPRAGE data. Abbreviations: GM, grey matter. ns indicates a non-significant difference and * indicates a significant difference at the pFDR < 0.05 level.


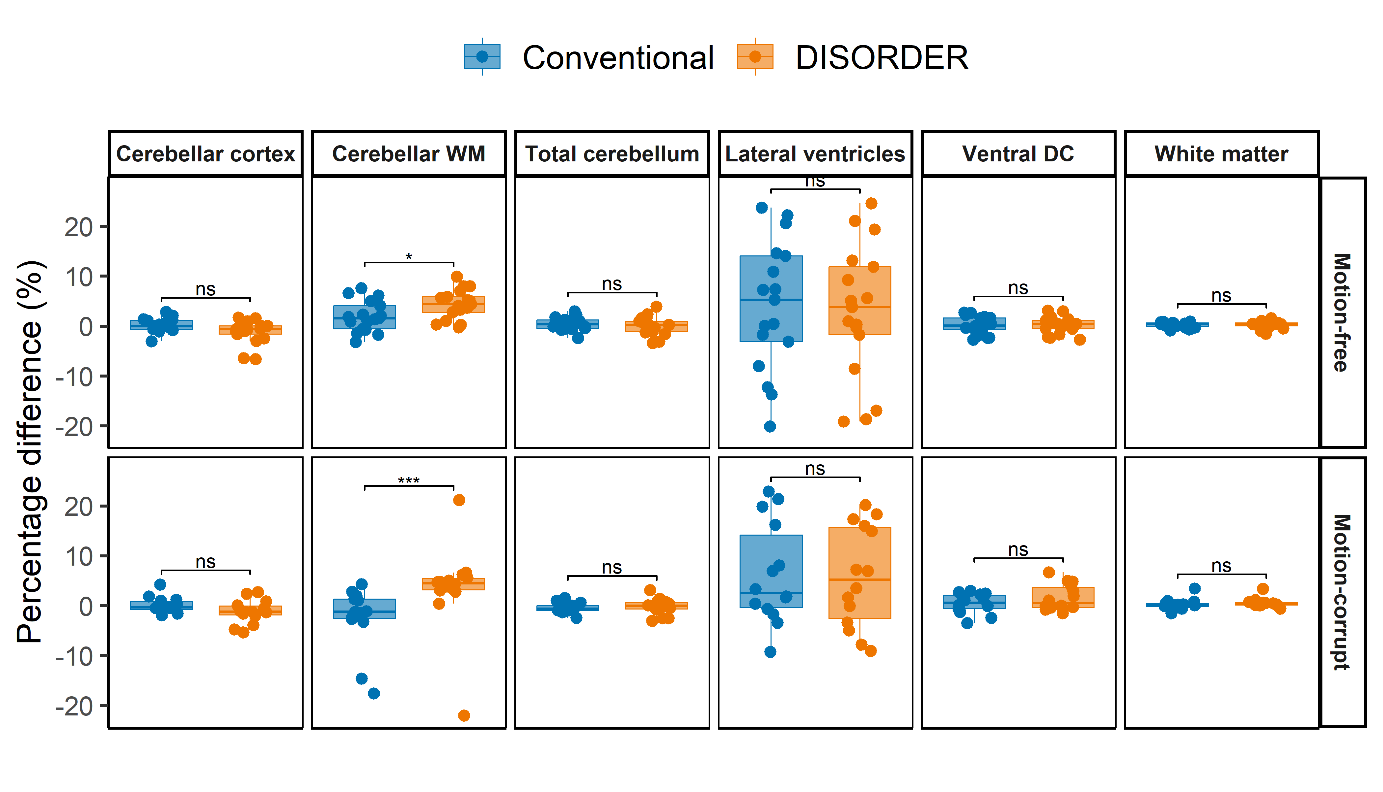


**Supplementary Figure 6.** Percentage difference between left and right regional brain volumes, shown separately for DISORDER and (i) motion-free and (ii) motion-corrupt conventional MPRAGE data. Abbreviations: DC, diencephalon; WM, white matter.ns indicates a non-significant difference and ** indicates a significant difference at the pFDR < 0.01 level.
